# A deep learning methodology for fully-automated quantification of calcific burden in high-resolution intravascular ultrasound images

**DOI:** 10.1007/s10554-025-03583-8

**Published:** 2025-12-27

**Authors:** Xingwei He, Mohamed O. Mohamed, Nathaniel Yu Jian Ng, Thamil Kumaran, Retesh Bajaj, Nathan Angelo Lecaros Yap, Emrah Erdogan, Gonul Zeren, Anthony Mathur, Ahmet Emir Ulutas, Bo Gao, Yaojun Zhang, Andreas Baumbach, Jouke Dijkstra, Christos V. Bourantas

**Affiliations:** 1 Department of Cardiology, Barts Heart Centre, Barts Health NHS Trust, London, UK; 2 Division of Cardiology, Department of Internal Medicine, Tongji Hospital, Tongji Medical College, Huazhong University of Science and Technology, Wuhan, China; 3 Institute of Health Informatics, University College London, London, UK; 4 Centre for Cardiovascular Medicine and Devices, William Harvey Research Institute, Queen Mary University, London, UK; 5 Department of Cardiology, Faculty of Medicine, Yuzuncu Yil University, Van, Turkey; 6 Department of Cardiology, Affiliated Hospital of Hubei, Suizhou Central Hospital, University of Medicine, Suizhou, China; 7 Department of Cardiology, Xuzhou Third People’s Hospital, Xuzhou, China; 8 Division of Image Processing, Department of Radiology, Leiden University Medical Center, Leiden, The Netherlands

**Keywords:** Coronary artery calcium, Coronary artery disease, Intravascular ultrasound, Machine learning

## Abstract

Quantification of the calcific burden is valuable in percutaneous coronary intervention (PCI) planning and in research to assess its changes after pharmacotherapies targeting plaque progression. In intravascular ultrasound (IVUS) images this analysis is currently performed manually and time consuming. To overcome these limitations, we introduce a deep-learning (DL) method for seamless detection of the calcific tissue. IVUS images from 197 vessels were analysed by an expert who identified the presence of calcium, and these estimations were used to train a DL model for fast detection of calcific deposits. The output of the model was tested in a set of 30 vessels against the estimations of the two experts. Comparison was performed at a frame-, lesion- and segment level. In total 26,211 frames were included in the training and 5,138 in the test set. The estimations of the DL method for the presence of calcium were similar to the experts (kappa 0.842 and 0.848, *p* < 0.001), while the correlation between the DL approach and the two experts for the arc of calcium (0.946 and 0.947, *p* < 0.001) and calcific area (0.745 and 0.706, *p* < 0.001) were high. Lesion- (0.971 and 0.990, *p* < 0.001) and segment-level analysis (0.980 and 0.981, *p* < 0.001) demonstrated a high correlation between the method and the two experts for calcific burden. The proposed DL method is able to accurately detect the calcific tissue and quantify its burden. These features render it useful in research and are expected to facilitate its application in the clinical workflows to guide PCI.

**Supplementary Information:**

The online version contains supplementary material available at 10.1007/s10554-025-03583-8.

## Introduction

Coronary calcification is a marker of advanced coronary artery disease (CAD) and poses a challenge in the percutaneous treatment of obstructive CAD [1, 2]. Intravascular imaging and in particular intravascular ultrasound (IVUS) is regarded today as the ideal modality for the detection of calcific tissue in the coronary arteries. Cumulative data has shown that it outperforms coronary angiography and is more sensitive than optical coherence tomography (OCT) in detecting calcific tissue and differentiate it from the lipid when this is deeply embedded [3–5]. The quantification of the calcific burden in IVUS images has recently attracted attention as serial IVUS imaging studies have shown that specific drugs can promote calcific tissue deposition and change coronary plaque phenotypes [6, 7]. In addition, recent evidence have shown that accurate assessment of calcific tissue morphology and extent is useful in percutaneous coronary intervention (PCI) planning and in particular in identifying lesions that will require preparation with debulking techniques to ensure optimal stent expansion [8–10]. 

Several methodologies over recent years have been developed that rely either on the radiofrequency analysis of the reflected IVUS signal or the pixel intensity of the plaque or even on the use of machine learning algorithms to identify calcific extent [11–19]. However, none of these have been widely adopted in clinical practice and research either because they have been proven inaccurate or because they have not been extensively validated or because they have not been incorporated in user-friendly software. Therefore, calcific tissue annotation is currently performed manually; this process, however, is time-consuming and can lead to erroneous estimations when analysis is undertaken by inexperienced operators.

The present study was designed to address this unmet need and introduce a fully automated deep-learning (DL) method for accurate quantification of calcific burden in IVUS images that has been incorporated in a user-friendly software and has been extensively validated against expert analysts.

## Methods

### Study population

We retrospectively analysed IVUS imaging data from patients that underwent 3-vessel IVUS imaging as part of two clinical studies. The first study enrolled 70 patients with a chronic coronary syndrome and obstructive disease on coronary angiography (NCT03556644) [20]. All recruited patients underwent CTA followed by coronary catheterization, three-vessel near-infrared spectroscopy (NIRS)-IVUS imaging and PCI or coronary physiology assessment when this was indicated. The second study included 20 patients with a chronic coronary syndrome that were listed for CTA and positron emission tomography imaging with the radiotracer Gallium-68 DOTATATE and then for coronary angiography and 3-vessel NIRS-IVUS and OCT imaging in order to examine the performance of hybrid non-invasive imaging in assessing vascular pathology using multimodality intravascular imaging as the reference standard (NCT04205111). The study was conducted in line with the Declaration of Helsinki and approved by the local research ethics committee. All participants provided written informed consent before study enrolment.

### NIRS-IVUS data acquisition

Only the NIRS-IVUS imaging data collected in the above studies were included in the present analysis. NIRS-IVUS imaging was performed in all patients before PCI in their 3 major epicardial vessels and large side branches (diameter ≥ 2 mm) using a 2.4 F Makoto™ NIRS-IVUS Imaging System (Infraredx a Nipro Company, Burlington, MA, USA). Intracoronary nitroglycerine was first injected and then the probe was advanced to the distal vessels and withdrawn using an automated pull-back device at a speed of 0.5 mm/s. The NIRS-IVUS images were acquired at 30 frames per second and stored in a DICOM format.

### IVUS segmentation

IVUS segmentation was performed in the end-diastolic frames - retrospectively identified using a well-validated DL algorithm - by a well-trained analyst with an established reproducibility [21, 22]. In these frames the lumen and external elastic membrane (EEM) border were manually annotated, in line with the previous published guidelines for IVUS analysis, as previously described [22, 23]. Analysis was performed using the QCU-CMS software (Version 4.69, Leiden, University Medical Center, Leiden, The Netherlands). The EEM border in frames portraying extensive calcification (>90^o^) was extracted using an automated interpolation approach that takes into account the EEM border in the arc that is visible and the EEM annotations in adjacent frames.

**Fig. 1:**
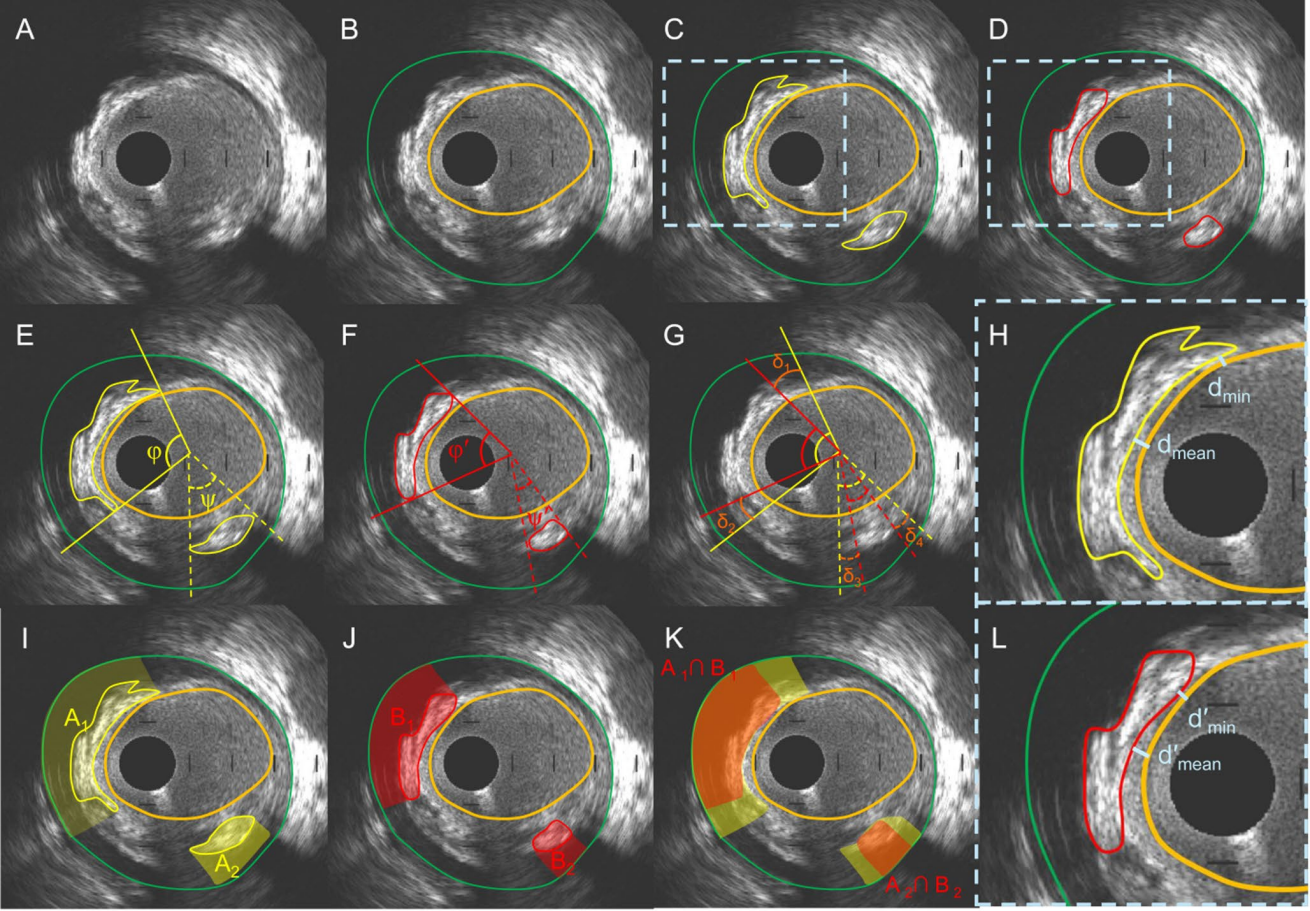
Methodology developed to compare calcium annotations of the experts and the DL method in an IVUS frame (A). The lumen and EEM borders are shown in panel (B) while panel (C) and (D) illustrate the annotations of the DL method and the expert for the calcific tissue. The angles φ, φ′, ψ and ψ′ indicate the circumferential extend of the annotated calcium in panels (E) and (F) while panel (G) portrays the angle differences δ_1_, δ_2_, δ_3_ and δ_4_ in the lateral extremities of the annotated calcific borders. Panel H and I portray a magnified view of the estimations of the DL method and the expert for the calcific deposit showing the minimum and mean distance estimations between the annotated borders (d_min_, d_mean_, d′_min_ and d′_mean_) and the lumen borders. Panel I and J illustrates the calcific tissue area estimations of the DL method (A and B) and the expert (A′ and B′) that are defined by the most proximal to the lumen calcific border its lateral extremities and the EEM border and K portrays then non-overlapping area analysis with the area shown in orange corresponding to the overlapping estimations (A∩A′ and B∩B′)

In the annotated IVUS frames an expert analyst identified the presence of calcific tissue – defined as areas brighter than the reference adventitia with acoustic shadowing behind with or without reverberations [23–25]. In each IVUS frames the calcific area was defined by the bright most proximal to the lumen calcific border and its lateral extend whereas its distal border was assumed that it corresponded to the EEM border (Fig. 1) [13, 26]. 

All the NIRS-IVUS data collected in the first study was used to train a DL classifier for the automated detection of calcific tissue, while the NIRS-IVUS data collected in the second study to test its performance. In the latter dataset – that consisted of 30 vessels – analysis was performed by another expert analyst twice (within a 3months interval so as to report its intra-observer variability) and by a third expert to assess the inter-observer variability.

The annotated calcific tissue was plotted in spread out plots with the x axis indicating the longitudinal position of the calcific tissue and the y axis its circumferential distribution and this was used to define the calcific burden index (CaBI) that was computed as the number of pixels corresponding to the presence of calcium divided by the total number of pixels multiplied by 1000 (Supplementary Fig. 1) [27]. 

### Deep-learning model development for calcific tissue detection

**Fig. 2:**
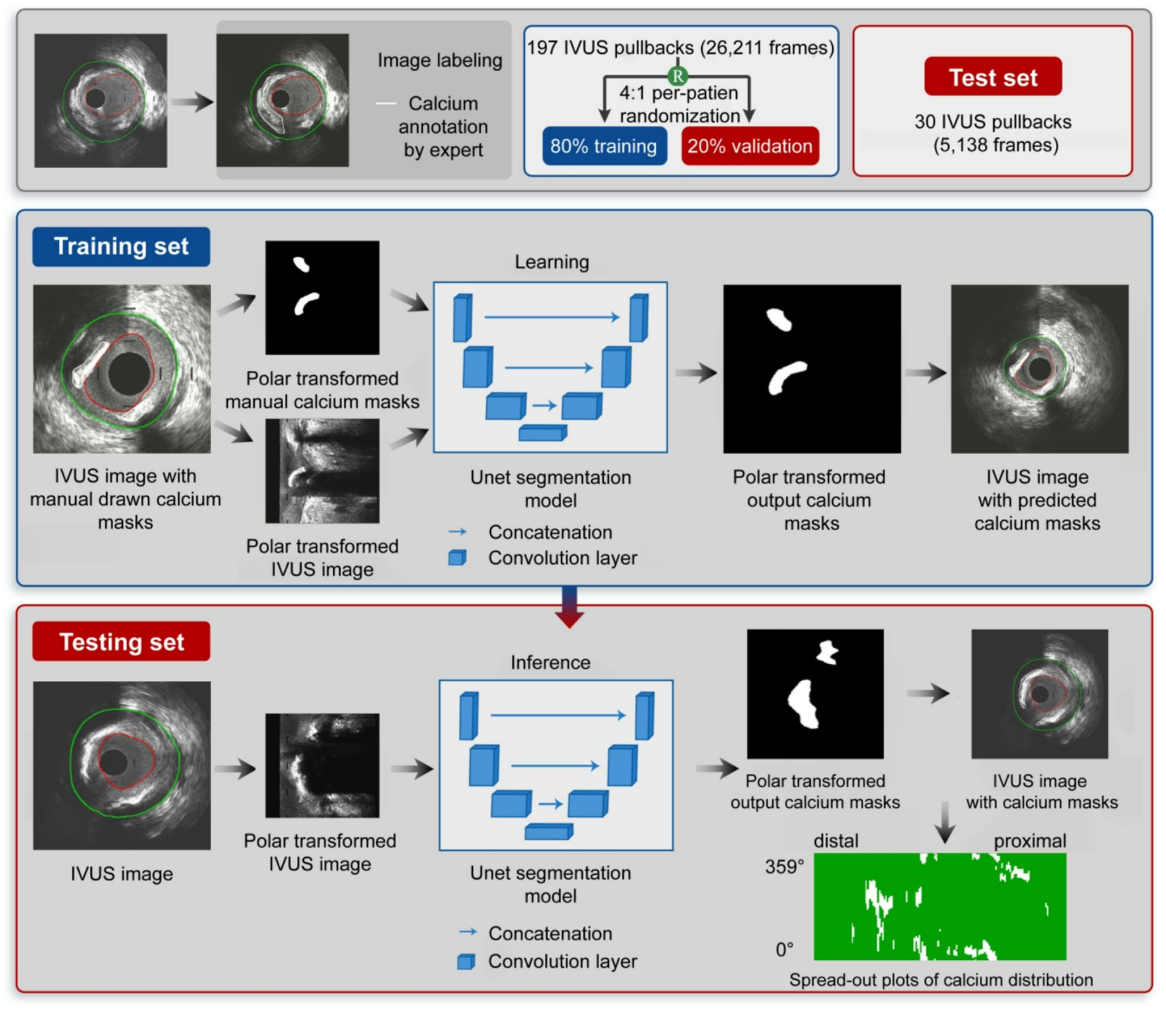
Schematic representation of the DL-methodology introduced for accurate detection of calcific tissue

This study developed a U-Net deep learning model to detect calcific deposits in IVUS images. We choose for training a U-Net model as this can be trained in small datasets, it is computationally inexpensive and fast allowing analysis of large datasets in real-time and it can be easily integrated in commercially available solutions allowing its broad application in clinical practice and research. Original frames (480 × 480) were converted to polar coordinates (240 × 240) and normalized (0–1), ensuring calcium-related shadowing always appeared in the same direction, simplifying training. The masks that have been drawn by the experts were also transformed into the same polar coordinate system and used as ground truth for training. These image data were used as an input to train the DL classifier; the output of the DL model was again in polar coordinates which was then transformed back into the original Cartesian space and mapped onto the IVUS images (Fig. 2**).** Post processing was performed and very small regions ≤ 50 pixels were removed as well as regions identified outside the lumen-vessel regions.

The Unet model consisted of three contracting layers and three expansions layers. The contracting path is a convolutional network that consists of two convolutions, each followed by a rectified linear unit and a max pooling operation. During the contraction, the spatial information is reduced, while feature information is increased. The expansive pathway combines the feature and spatial information through a series of up-convolutions and concatenations with high-resolution features from the contracting path.

Training used 26,211 frames (80% training, 20% validation), 50 epochs, learning rate 10 e-5, and binary cross-entropy loss. Loss curves showed no overfitting. Postprocessing removed regions ≤ 50 pixels and those outside the lumen. The method was integrated into QCU-CMS software for fully automated calcium segmentation [21]. 

### Statistical analysis

Numerical variables were tested for normality with the Kolmogorov-Smirnov test. Normally distributed data are presented as mean ± SD, non-normal as median (IQR), and categorical as counts and percentages, compared using the Chi-squared test.

To evaluate the DL-method, both quantitative and qualitative analyses were performed. At the frame level, we reported the prevalence, arc and area of calcium, as well as mean/minimum lumen–calcium distances (Fig. 1). Agreement on calcium presence between experts and the DL-method was assessed with the Kappa statistic, and diagnostic performance (sensitivity, specificity, positive predictive value, negative predictive value, accuracy) was calculated using expert annotations as reference. Pearson correlation (r) was performed to examine the association between the estimations of the analysts and the DL method for the calcific arc and area and the average and minimum distance between the lumen and the calcific border.

Lesions were defined in NIRS-IVUS as ≥ 3 consecutive frames with plaque burden ≥ 40% and classified as calcific-rich when calcium arc ≥ 90° [27]. The sensitivity specificity, positive and negative predictive value and accuracy of the DL method to detect calcific-rich lesions was reported using the two experts’ estimations as a reference standard. Moreover, we compared the estimations of the DL-method and the experts for the CaBI in the studied lesions and segments.

Qualitative analysis was performed at a frame-level and included comparison of the circumferential distribution of the calcific tissue and overlapping area analysis of the estimations of the experts and the DL-method. To estimate the agreement of the experts and the DL-method for the circumferential distribution of the calcific tissue, we estimated the mean-angle difference between the lateral extremities of the annotated calcium, while the Dice index was used for the overlapping area analysis (Fig. 1). Spearman correlation was applied to compare the Dice indices, among which there were linear relationships, reported as Spearman’s *rho* with corresponding p-values for which a significance was set at 0.05.

To examine whether the DL-methodology is as accurate as the two experts in detecting calcific tissue we computed the Williams Index (WI) and its 95% confidence interval for all the measured metrics [28]. This index was developed to compare the agreement between a method of unknown performance (i.e., the DL-methodology) and established analysis approaches (i.e. the two experts) with the agreement of the established approaches. A WI close to one indicates that the differences between the estimations of the DL-method and the experts are not larger than their inter-observer variability, indicating that the DL-method method is as accurate as the experts.

## Results

### Studied patients

Data from 78 patients were included in the analysis; the NIRS-IVUS sequences from 65 patients (197 vessels, 26,211 frames) were used to train the DL-model and from 13 patients (30 vessels, 5,138 frames) to test its performance. The baseline demographics of the studied patients are listed in Supplementary Table 1.

### Quantitative analysis results

In the test set, calcific tissue was detected by the DL method in 1,320 frames, in 1,182 frames by the 1 st expert in the 1 st analysis, in 1,183 frames by the same expert in the 2nd analysis and in 1,311 frames by the 2nd expert. The Kappa statistics between the estimations of the DL method and the experts for the presence of calcium was high and ranged between 0.842 and 0.848 (*p* < 0.001 for both analysts), while for the presence of calcium ≥ 90^o^ it ranged between 0.752 and 0.802 (*p* < 0.001 for both analysts) (Table 1). The sensitivity specificity positive and negative predictive value of the DL solution to detect calcific tissue was 0.94, 0.94, 0.84 and 0.98 while the accuracy was 0.94.

**Table 1:** Kappa values between the estimations of the DL methodology and the experts and between the experts for the detection of the calcific tissue in the analysed frames and the detected lesions

	Frame-level analysis		Lesion level analysis	
	Presence of calcium	*p*-value	Calcium ≥ 90^o^	*p*-value
DL vs. Expert 1 First Analysis	0.842	< 0.001	0.752	< 0.001
DL vs. Expert 2	0.848	< 0.001	0.802	< 0.001
Expert 1 first analysis vs. Expert 2	0.936	< 0.001	0.896	< 0.001
Expert 1 first vs. second analysis	0.888	< 0.001	0.901	< 0.001

DL, deep learning

**Fig. 3:**
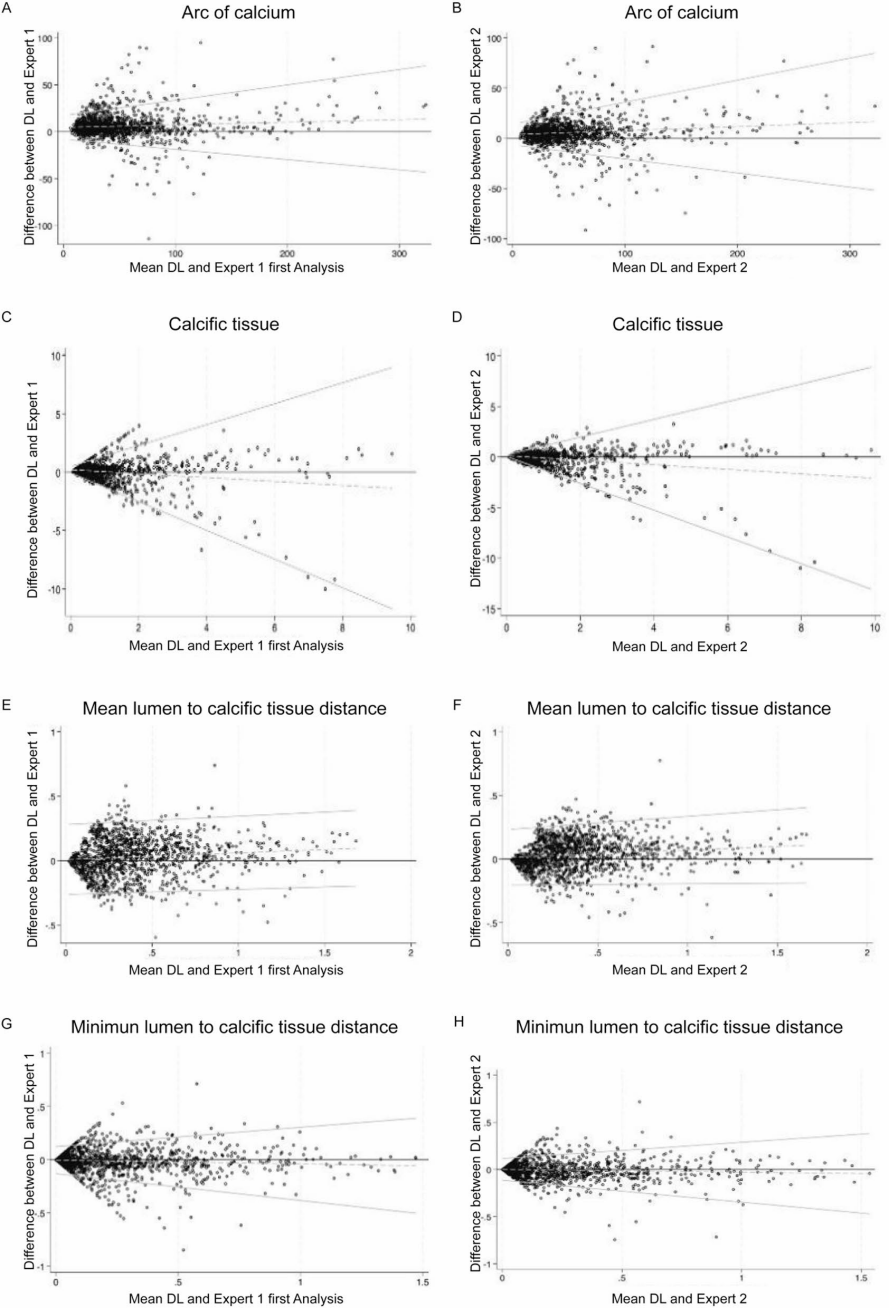
Bland-Altman plots displaying the bias and limits of agreement between the estimations of the DL method and the 1 st and 2nd expert for the arc of calcific tissue (A and B) for the calcific area (C and D) and for the mean (E and F) and minimum (G and H) distance between the lumen and and calcific borders

A high correlation was noted between the estimations of the DL method and the experts for the arc of calcium, and for the mean and minimum distance of the calcific tissue from the lumen border, while the correlation between the DL estimations and the experts for the calcific areas was moderate. Bland-Altman analysis also confirmed a small bias and narrow limits of agreement between the estimations of the two experts and the DL method (Table 2; Figs. 3).

**Table 2:** Pearson correlation coefficient and mean difference and standard deviation between the estimations of the DL and the experts for the Arc of the calcific tissue the mean and minimum lumen to calcific tissue distance and calcific area

	Pearson correlation	*p*-value	Mean difference ± SD
Frame-level analysis	Coefficient		
**Arc of calcium**			
DL vs. Expert 1 first analysis (degrees)	0.946	< 0.001	5 (1.5)
DL vs. Expert 2 (degrees)	0.948	< 0.001	4.5 (0.5)
Expert 1 first analysis vs. Expert 2 (degrees)	0.954	< 0.001	0.5 (2)
Expert 1 first vs. second analysis (degrees)	0.971	< 0.001	3.5 (1.5)
**Calcific tissue area**			
DL vs. Expert 1 first analysis (mm^2^)	0.745	< 0.001	0.069 (0.180)
DL vs. Expert 2 (mm^2^)	0.706	< 0.001	0.029 (0.258)
Expert 1 first analysis vs. expert 2 (mm^2^)	0.898	< 0.001	0.098 (0.078)
Expert 1 first vs. second analysis (mm^2^)	0.945	< 0.001	0.032 (0.022)
**Mean lumen to calcific tissue distance**			
DL vs. Expert 1 first analysis (µm)	0.908	< 0.001	28 (15)
DL vs. Expert 2 (µm)	0.876	< 0.001	38 (36)
Expert 1 first analysis vs. expert 2 (µm)	0.932	< 0.001	10 (21)
Expert 1 First vs. second analysis (µm)	0.885	< 0.001	15 (25)
**Minimum lumen to calcific tissue distance**			
DL vs. Expert 1 first analysis (µm)	0.930	< 0.001	14 (38)
DL vs. Expert 2 (µm)	0.906	< 0.001	2 (9)
Expert 1 first analysis vs. Expert 2 (µm)	0.923	< 0.001	12 (29)
Expert 1 First vs. second analysis (µm)	0.944	< 0.001	16 (14)
**Lesion-level analysis**			
*CaBI*			
DL vs. Expert 1 first analysis	0.971	< 0.001	16.5 (14.9)
DL vs. Expert 2	0.990	< 0.001	12.9 (14.9)
Expert 1 first analysis vs. expert 2	0.998	< 0.001	5.3 (7.6)
Expert 1 first vs. second analysis	0.991	< 0.001	3.6 (1.3)
**Segment-level analysis**			
*CaBI*			
DL vs. Expert 1 first analysis	0.980	< 0.001	1.5 (2.2)
DL vs. Expert 2	0.981	< 0.001	1.9 (1.4)
Expert 1 first analysis vs. Expert 2	0.997	< 0.001	1 (0.2)
Expert 1 first vs. second analysis	0.993	< 0.001	0.4 (0.8)

CaBI, calcific burden index, DL, deep learning; SD, standard deviation

In the test group 60 lesions were identified; based on the estimations of the experts, 22 lesions were calcific-rich with an arc of calcium ≥ 90^o^ (24 according to the 1 st expert in the 2nd analysis) while the DL method identified 25 calcific-rich lesions. The sensitivity, specificity, positive, negative predictive value and the accuracy of the DL-method to detect calcific-rich lesions was excellent, estimated at 95.5%, 89.5%, 84.0%, 97.1% and 91.7% respectively.

**Fig. 4:**
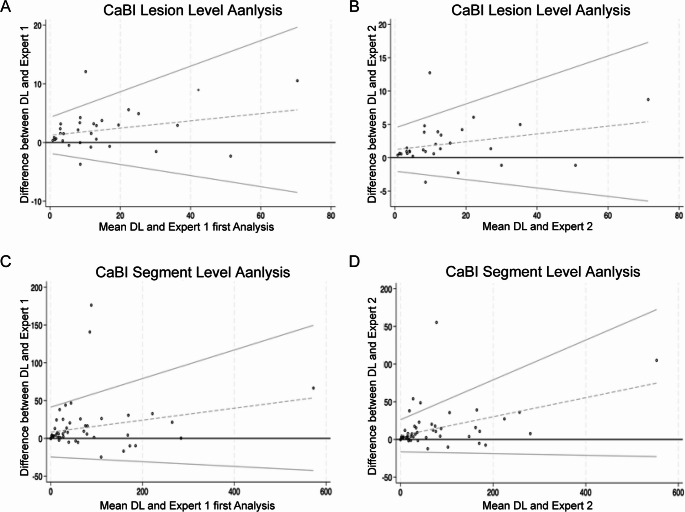
Bland-Altman plots displaying the bias and limits of agreement between the estimations of the DL method and the 1 st and 2nd expert for the CaBI estimated from the lesion- (A and B) and segment level analysis (C and D)

A high correlation was noted for the CaBI estimations between the DL method and the experts in the studied lesions and segments (on all occasions ≥ 0.971), while Bland-Altman analysis showed a small bias and narrow limits of agreement between DL estimations and expert analysts (Table 2; Figs. 4).

### Qualitative analysis results

The mean difference between the lateral extremities of the calcific deposits detected by the DL method and the experts was close to 0 and the standard deviation was small, while the Dice index was high for the non-overlapping area analysis measured 0.915 between the estimations of the DL method and the 1 st and 0.916 between the DL method and the 2nd expert.

Manual detection of the calcific tissue in a representative 5000-frame vessel took approximately 1.5 h; conversely, the DL-methodology was able to segment this vessel in < 1 min using a desktop PC with an Intel Core i5 processor and 8 GB of RAM.

### Intra and interobserver variability

A high inter- and intra-observer agreement was also noted for the annotations of the two experts. The kappa for the presence of calcific tissue was 0.936 and 0.888 respectively. and the correlation was excellent for the arc of calcium, the measured calcific area and for the mean and minimum distance between the lumen border and the calcific tissue. The Bland-Altman analyses showed low bias and narrow limits of agreement between the estimations of the 1 st expert and 2nd expert and for the two analyses of the 1 st expert for all the studied variables (Table 2; Figs. 3 and Supplementary Fig. 2).

Lesion-level and segment-level analysis also confirmed a high-inter and intra observer reproducibility for the measured CaBI, while qualitative analysis demonstrated small differences between the circumferential distribution of the calcific tissue and a high Dice index between the two experts and the two analyses of the 1 st expert (Table 3; Figs. 4 and Supplementary Fig. 3).

**Table 3:** Qualitative assessment of the agreement of the estimations of the DL method and the experts and expert analysts

	Mean angle difference of the circumferential distribution of the calcific tissue	Dice index
DL vs. Expert 1 First Analysis	0.02 ± 8.5	0.534
DL vs. Expert 2	−0.06 ± 8	0.524
Expert 1 First Analysis vs. Expert 2	−0.1 ± 5.5	0.808
Expert 1 First vs. Second Analysis	−0.2 ± 7.6	0.771

### Williams index

The Williams Indices are listed in Table 4. The index was < 1 in most of the studied variables apart from the minimum distance between the lumen border and the calcific tissue border where it was 1.613 indicating that the DL method enables more accurate assessment of this metric. The minimum Williams Index was reported for the CaBI at a segment level and was 0.347 (Table 4).

**Table 4:** Williams index assessing the agreement of the DL method and the expert estimations

	Williams Index	CI
Frame-level analysis		
Calcific angle	0.593	0.591–0.594
Average lumen to calcific tissue distance	0.751	0.750–0.752
Minimum lumen to calcific tissue distance	1.631	1.624–1.637
Calcific area	0.528	0.527–0.529
Total angle difference	0.600	0.599–0.602
Angle difference of the circumferential location of calcific tissue	0.592	0.591–0.593
Dice index	0.915	0.915–0.916
**Lesion-level analysis**		
CaBI	0.506	0.460–0.552
**Segment-level analysis**		
CaBI	0.347	0.317–0.377

CaBI, calcific burden index

## Discussion

In this study, we introduced, a DL approach for the detection of calcific tissue in high-resolution NIRS-IVUS. We found that the DL method enables (1) accurate detection of calcific tissue (2) reliable assessment of its circumferential extent and area (3) precise identification of its location within the plaque (4) accurate classification of plaques as calcific or non-calcific and (5) provides estimations that are similar to the expert analysts for the CaBI in lesion- and segment-level analysis.

Cumulative data have underscored the value of intravascular imaging in guiding PCI and today both IVUS and OCT have IA indication in the European Society of Cardiology and American College of Cardiology guidelines for the treatment of complex lesions [29–31]. Despite the robust evidence supporting its routine use in the clinical practice both modalities have limited applications; in the UK according to the BCIS audit data only ≈ 25% of the PCIs are performed under intravascular imaging guidance. The increased cost, the time needed for image acquisition and analysis and the lack of expertise are considered today the main reasons for the restricted applications of intravascular imaging in daily practice.

To overcome these limitations several software solutions have been introduced for automated segmentation and characterisation of plaque composition of intravascular imaging data nevertheless only two of them – one for each modality – have reached the clinical practice [32]. The Ultreon software developed by Abbott Vascular (Santa Clara, CA) for OCT analysis enables not only detection of the lumen borders and characterisation of lesion severity but also quantification of the calcium extent that is important in procedure planning and seems to predict stent apposition results [8, 10]. Conversely the AVVIGO™ lesion analysis system introduced by Boston Scientific (Marlborough, MA) is limited only to the assessment of the lumen and vessel wall dimensions and does not give information about calcium extent – therefore this useful information is not available int the current IVUS workflows.

Over the recent years several methodologies have been developed for characterising plaque composition on IVUS imaging and quantifying tissue types however all of them have significant pitfalls. The methods that rely on radiofrequency analysis of the IVUS backscatter signal have been proven unreliable for quantifying the necrotic core and have been withdrawn from the market, except for the integrated backscatter analysis, which is limited to use in Japan [11, 12, 33]. Methods that rely on the pixel intensity of the IVUS signal – the so called echogenicity – have been proven effective in assessing vessel wall response following treatment with endovascular devices [34] and provide information about plaque components with however limited accuracy and therefore they have a restricted use in research [12, 13, 35]. We have recently introduced a method that takes into account the distribution of the NIRS signal and the pixel intensity to characterise tissue types that was validated against histology with promising results [13]. However, testing was performed in only 62 calcific deposits and although the efficacy of the method to identify calcium was high its performance in estimating its extend was only moderate. Finally, the methods that have been presented in the literature and rely either on the pixel intensity analysis or on DL solutions to quantify the calcific component have either been tested only in a small number of frames against the estimations of expert analysts [17, 36–38] or of virtual histology [16, 19, 39] or they have been tested at scale without however the use of a thorough methodology that will enable evaluation of the location – depth and circumferential extent – of the calcific tissue in the plaque [15, 18] and have not been incorporated in a user-friendly platform that would allow their broad use in research.

This study overcomes the above limitations and introduces a fully automated solution for the detection of calcific tissue in high-resolution IVUS images. In contrast to previous reports, the presented DL-method was trained in a large dataset of >25,000 annotated frames, that included diseased and disease-free segments to avoid bias, using the estimations of expert analyst as a reference standard that appear to have a high accuracy in identifying calcific tissue in histology studies [11, 40]. Testing was performed in 30 vessels, 5,138 frames, allowing assessment of its performance at a lesion- and segment-level and including a qualitative analysis focusing on the performance of the DL method to detect the calcific tissue location in the plaque.“.

We found that the DL method was able to differentiate with a high accuracy, frames portraying calcific tissue from those without calcium and that our approach allowed not only reliable quantification of the calcific tissue but also evaluation of its circumferential distribution and depth. Moreover, lesion-level and segment-level analysis demonstrated that it had a high diagnostic performance in detecting calcific-rich plaques and that it provided estimations about the calcific extent in the studied lesions and segments that were similar to the estimations of the expert analysts. Despite the fact that there was a high agreement between the annotations of the DL method and the experts the Williams Index was moderate for most of the studied metrics and bellow 1 – apart from the minimum distance between the lumen border and the calcific tissue – indicating that the DL method is less reliable than the experts in quantifying the calcific extent. This paradox should be attributed to the excellent inter and intra-observer variability, noted between the two expert analysts, that was smaller than the differences noted between the output of the experts and the DL method. Considering the high correlation and the small bias and limits of agreements between the DL method and the experts and the fact that this approach has been incorporated in a user-friendly software and is fast and fully reproducible we believe that it constitutes a useful tool in research. This method can be combined with DL methodologies that have been designed to detect the lumen and vessel wall borders in IVUS and an approach for the differentiation of the fibrotic from the lipid-core plaques in NIRS-IVUS to fully characterise plaque phenotypes and assess for changes in plaques components in serial IVUS studies evaluating the implications of novel pharmacotherapies on plaque morphology [13, 21, 41]. This approach can also be useful in the clinical practice and in PCI planning as it enables not only quantification of the calcific burden but also assessment of its circumferential extent and depth that seems to be predictors of suboptimal stent expansion [8, 10]. 

### Limitations

This study has several limitations that should be acknowledged. Firstly, training was performed using the estimations of expert analysts and not using histological annotations that constitute the reference standard in the assessment of plaque composition. Although expert analysts seem to have a high diagnostic accuracy in detecting the presence of calcium, the use of large histological data may improve the diagnostic performance of this approach. Secondly, testing in this study was performed in a large dataset including annotated IVUS frames from disease and disease-free segments, however, the number of the studied vessels and lesions analysed was relatively small. Moreover none of the studied lesions had circumferential calcification or an arc of calcium >270 for a 5 mm segment so as to examine the performance of our solution to identify lesions that has been associated with a high risk of stent under-expansion [8].Thirdly this method does not overcome the limitations of IVUS in the quantification of the calcific burden as it is unable to detect the distal border of the calcific tissue and measure its thickness. Finally, the algorithm was developed in data collected in a single centre study by a specific high-resolution IVUS probe; this can restrict the generalizability of our findings as it is unclear whether our DL method will be equally effective in data acquired by different IVUS systems.

## Conclusions

The DL-learning methodology introduced in this study for the detection of the calcific tissue has a high agreement with the estimations of expert analysts about the CaBI and is able to reliably detect its circumferential extent. The fact that it is fast, accurate and reproducible and that it has been incorporated in a user-friendly software renders it a useful tool in research in the characterization of plaque composition and has the potential for clinical applications to assist PCI planning.

## Supplementary Information

Below is the link to the electronic supplementary material.

Supplementary Material 1

## Data Availability

No datasets were generated or analysed during the current study.
