# Supplementary material for: A deep learning methodology for fully-automated quantification of calcific burden in high-resolution intravascular ultrasound images
Source: Int J Cardiovasc Imaging. 2025 Dec 27;42(1):87–98. doi: 10.1007/s10554-025-03583-8 (PMC12847118; doi:10.1007/s10554-025-03583-8)

**A deep learning methodology for fully-automated quantification of calcific burden in high-resolution intravascular ultrasound images**

**Supplement**

Xingwei He, MD^1,2^; Mohamed O. Mohamed, PhD^1,3,^ ; Nathaniel Yu Jian Ng, MBChB^1^; Thamil Kumaran, PhD^4^; Retesh Bajaj, MBChB^1,4^; Nathan Angelo Lecaros Yap, MBChB^1^; [Emrah Erdogan](https://pubmed.ncbi.nlm.nih.gov/?term=Erdogan+E&cauthor_id=33703857)^5^; MBChB PhD^1^; Gonul Zeren, MD^4^; [Anthony Mathur](https://pubmed.ncbi.nlm.nih.gov/?term=Mathur+A&cauthor_id=33703857), MD, PhD^1,4^; Ahmet Emir Ulutas, MD^4^; Bo Gao^6^, Yaojun Zhang, MD, PhD^7^; Andreas Baumbach, MD, PhD^1,4^;; Jouke Dijkstra, PhD^8^; Christos V Bourantas, MD, PhD^1,4,*^

^1^ Department of Cardiology, Barts Heart Centre, Barts Health NHS Trust, London, UK

^2^ Division of Cardiology, Department of Internal Medicine, Tongji Hospital, Tongji Medical College, Huazhong University of Science and Technology, Wuhan, China

^3^ Institute of Health Informatics, University College London, London, United Kingdom

^4^ Centre for Cardiovascular Medicine and Devices, William Harvey Research Institute, Queen Mary University London, UK

^5^ Department of Cardiology, Faculty of Medicine Yuzuncu Yil University Van, Turkey

^6^Department of Cardiology, Suizhou Central Hospital, Affiliated Hospital of Hubei University of Medicine, Suizhou, China

^7^ Department of Cardiology, Xuzhou Third People’s Hospital, Xuzhou, China

^8^ Division of Image Processing, Department of Radiology, Leiden University Medical Center, Leiden, The Netherlands

**Short title:** DL method for calcium detection in IVUS

**Supplementary Table 1.** Baseline demographics of the training and test set.

|  | **Training set (n=65)** | **Test set (n=13)** | **p-value** |
| --- | --- | --- | --- |
| Age, mean (years) | 61.42±8.22 | 61.69±5.99 | 0.227 |
| Gender (male) | 50 (76.9%) | 10 (76.9%) | 0.899 |
| Body mass index (kg/m**^2^**) | 29.48±6.60 | 28.73±4.21 | 0.953 |
| **Medical history** |  |  |  |
| Diabetes mellitus | 22 (33.8%) | 8 (61.5%) | 0.061 |
| Hypertension | 35 (53.8%) | 9 (69.2%) | 0.334 |
| Hypercholesterolaemia | 46 (70.8%) | 11 (84.6%) | 0.304 |
| Renal failure* | 50 (76.9%) | 9 (69.2%) | 0.555 |
| Previous ACS | 9 (13.8%) | 1 (7.7%) | 0.545 |
| Previous PCI | 14 (21.5%) | 1 (7.7%) | 0.247 |
| Previous cerebrovascular event | 6 (9.2%) | 0 (0.0%) | 0.254 |
| Medications |  |  |  |
| Aspirin | 63 (96.9%) | 13 (100%) | 0.650 |
| P_2_Y_12_-inhibitor | 59 (90.8%) | 11 (84.6%) | 0.387 |
| Statin | 61 (93.8%) | 13 (100%) | 0.645 |
| β-blocker | 46 (70.8%) | 12 (92.3%) | 0.156 |
| RAAS inhibitor | 33 (50.8%) | 11 (84.6%) | 0.037 |
| **Studied vessels** | **(n=197)** | **(n=30)** | **0.563** |
| LAD/diagonal branches | 67 (34.0%) | 8 (26.7%) |  |
| LCx/intermediate/obtuse marginal branches | 81 (41.1%) | 12 (40.0%) |  |
| RCA | 49 (24.9%) | 10 (33.3%) |  |

**Table footnote:** ACS, acute coronary syndrome; LAD, left anterior descending; LCx, left circumflex; PCI, percutaneous coronary intervention; RAAS, renin angiotensin aldosterone system; RCA, right coronary artery.

*Renal failure was defined as estimated glomerular filtration rate <90 mL/min/1.73m^2^.

**Supplementary Figure 1.**

Spread-out plots of the calcific tissue distribution estimated by the DL (A) method and the experts; panel (B) indicates the estimations of the 1^st^ expert, (C) the estimations of the 1^st^ expert when he repeated the analysis and (D) the estimations of the 2^nd^ expert. The cross-sectional images below the spread-out plot indicate the annotated calcific tissue by the DL method and the experts and the defined calcific area shown in semi-transparent white – while the remaining plaque is illustrated in semi-transparent green - at the location of the minimum lumen area (left) and the maximum calcific burden (right).


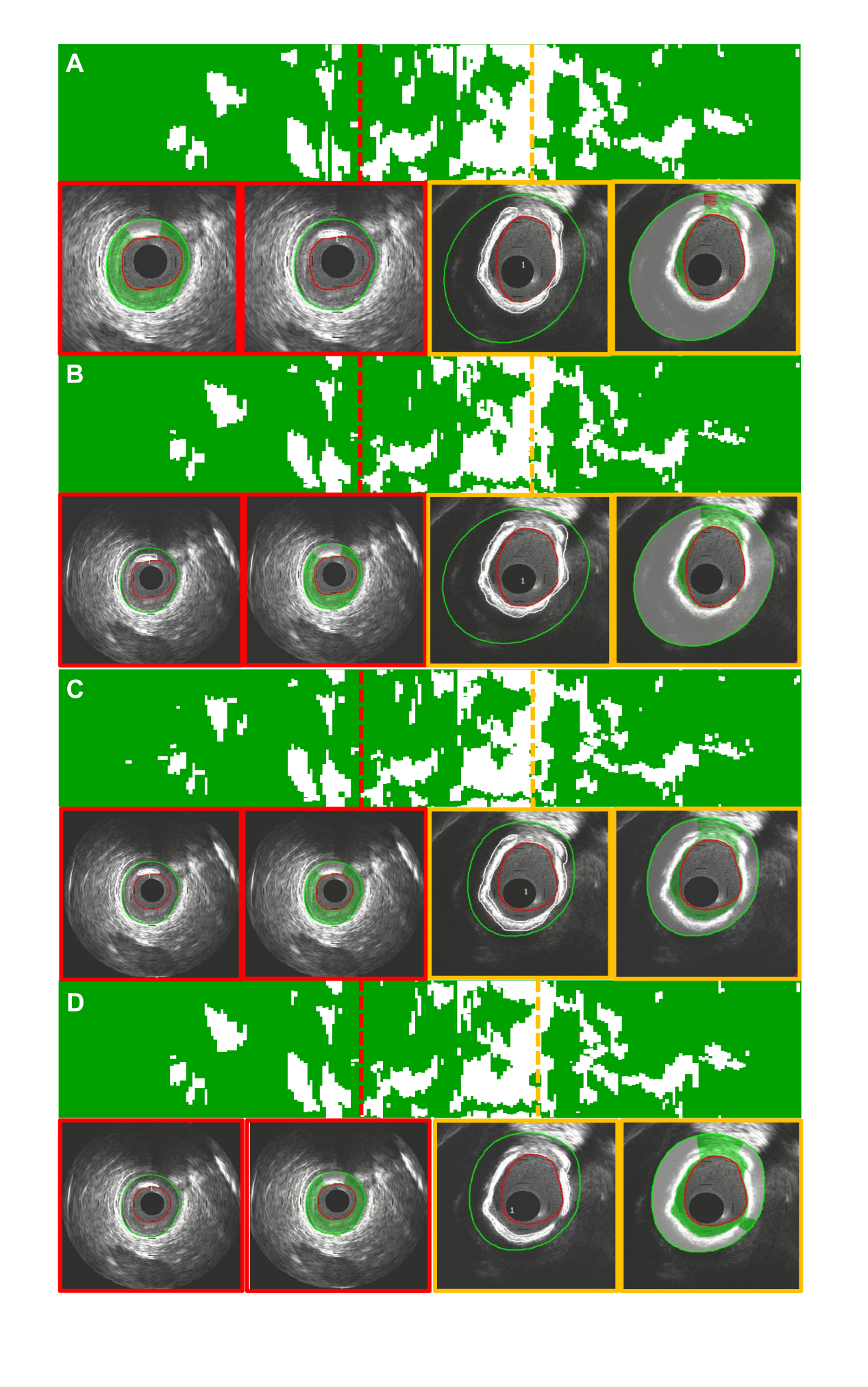


**Supplementary Figure 2.**

Bland-Altman plots displaying the low bias and narrow limits of agreement between the estimations of the 1^st^ expert and 2^nd^ expert and for the two analyses of the 1^st^ expert for the arc of calcific tissue (A and B) for the calcific area (C and D) and for the mean (E and F) and minimum (G and H) distance between the lumen and and calcific borders.


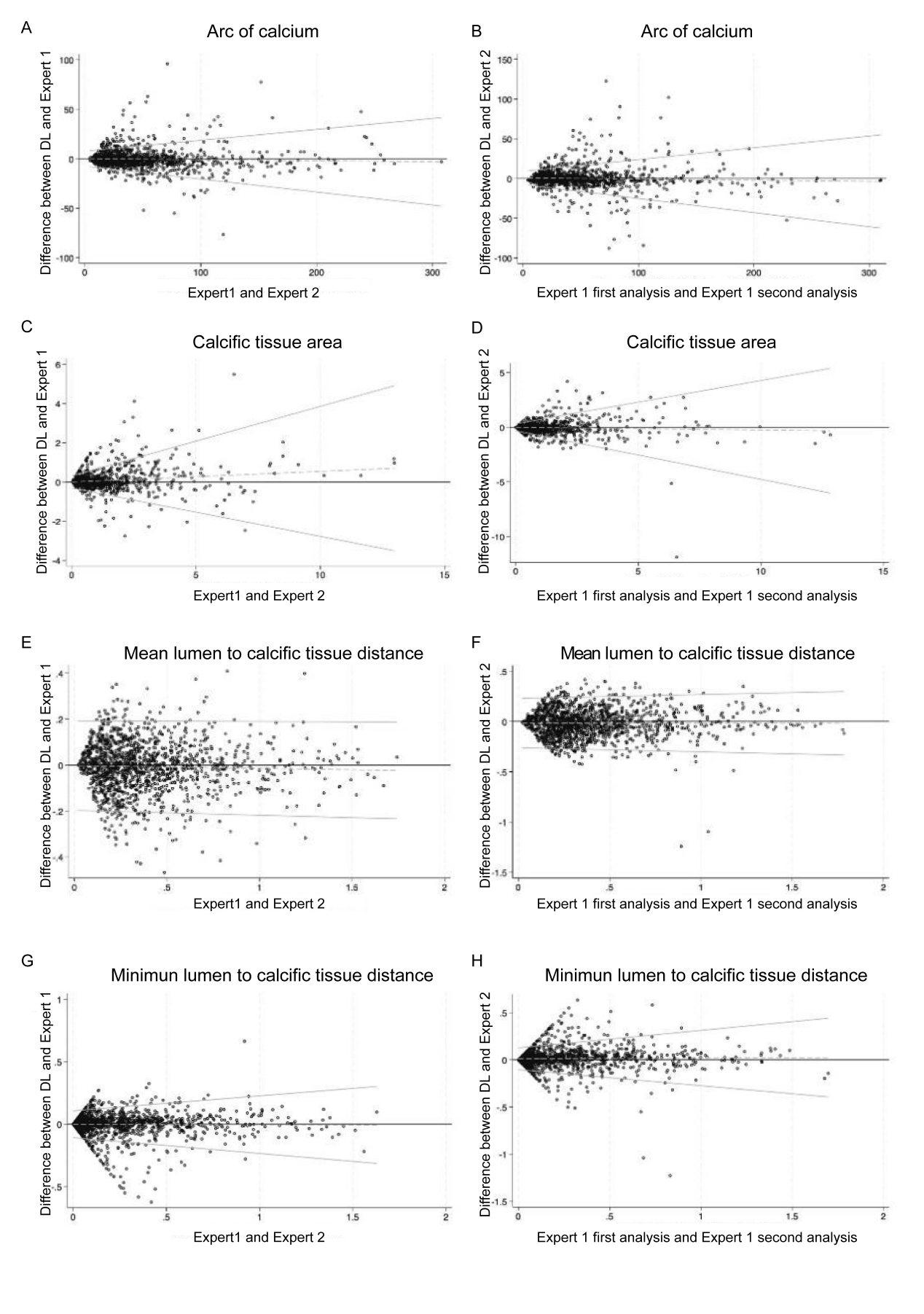


**Supplementary Figure 3.** Bland-Altman plots displaying the bias and limits of agreement between the estimations of the 1^st^ expert and 2^nd^ expert and for the two analyses of the 1^st^ expert for the CaBI estimated from the lesion- (A and B) and segment level analysis (C and D).


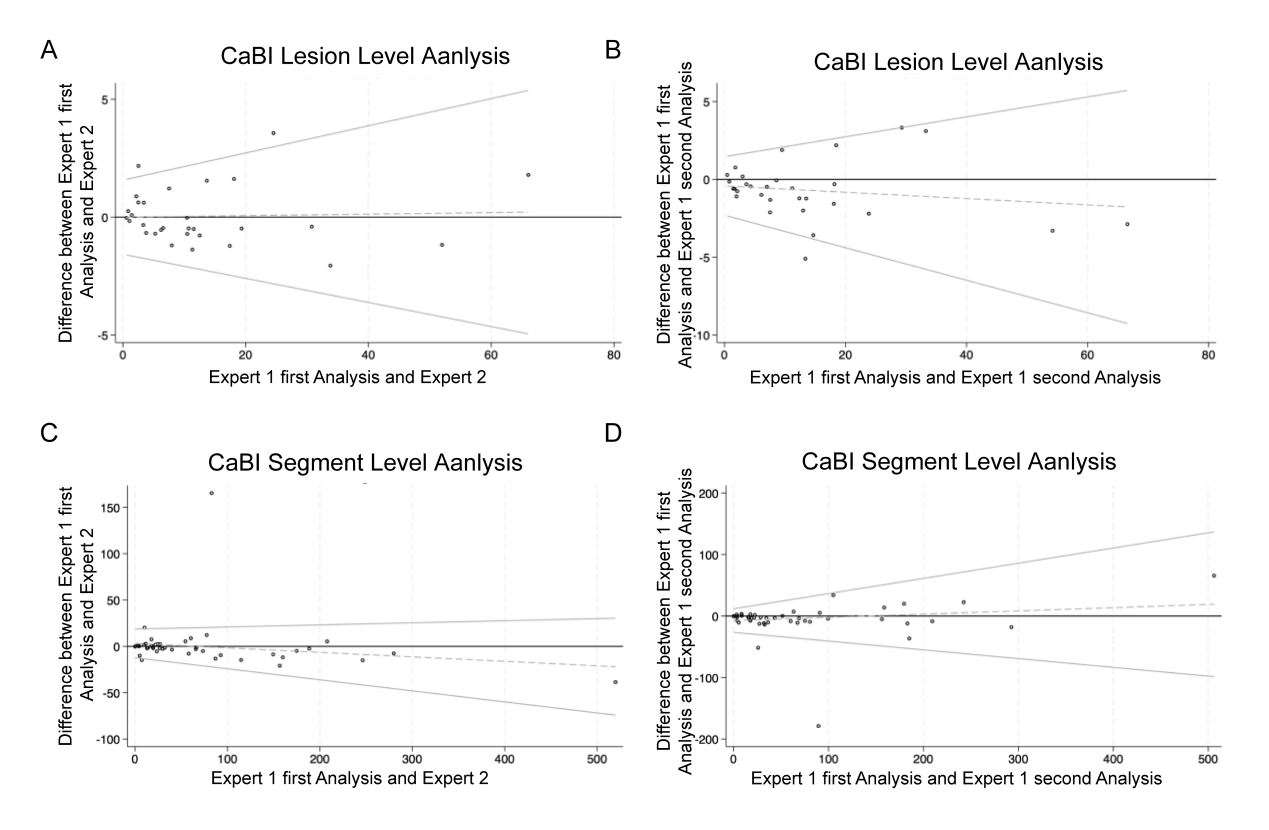

Supplement: Supplementary file 1 — Supplementary Material 1 [file 10554_2025_3583_MOESM1_ESM.docx]
